# Some Remarks on Porcelain Inlays

**Published:** 1904-07

**Authors:** Rea Proctor McGee

**Affiliations:** Denver, Colo.


### PORCELAIN INLAYS.

By Geo. N. Wasser, D. D. S., Cleveland, Ohio.

(Written for *The American Journal of Dental Science.*)

### SOME REMARKS ON PORCELAIN INLAYS.

By Rea Proctor McGee, M. D., D. D. S., Denver, Colo.

(Written for *The American Journal of Dental Science.*)

The porcelain inlay, in suitable cases, is the most perfect substitute for missing tooth structure that has yet been devised. In unsuitable cases it is a complete failure. It is indicated in fractures of the anterior teeth where less than half of the crown of the tooth is lost; in large cavities where gold would be unsightly; in cases where the patient is physically unable to stand the condensation of gold; in very frail teeth; and in cavities in which the pulp is nearly exposed and it is undesirable to devitalize, particularly in children's teeth.

The inlay is contra-indicated in small cavities upon the occlusal surface, or in any position where there is not sufficient bulk of porcelain to withstand the strain of mastication. The cavity must be so cut that the matrix can be removed without bending. The base of the cavity must be as nearly as possible at right angles to the walls so that the margin of the inlay will have a considerable bulk and not fade away to a feather edge. The heavier the edge of the inlay, the stronger it will be and more perfect the margin. The inlay will also be more easily set and the cement will retain it better.

The inlays that fail are usually the ones that were set in saucer-shaped cavities. The thin edge chips away, making an imperfect margin. The thinning of the edge also destroys the color effect, giving a whitish appearance near the margin.

I have always used the high fusing bodies and find them very satisfactory. I know nothing of the low fusing bodies, never having tried them.

After having prepared my cavity, with the margin as deep as possible, I swedge a matrix of very thin platinum foil with pellets of wet cotton, condensed with an amalgam plugger. Without trimming the matrix I place a little finely ground Close's body in the center of the depression and biscuit it. Then the matrix is approximately trimmed, returned to the cavity and re-burnshed, removed, trimmed closely and again fitted to the cavity. Then the matrix is carefully removed and filled with the colored porcelain mixed to approximate the tooth shade.

If the inlay is to restore a contour, or is of considerable size, I make a gutta percha model by filling the cavity with gutta percha and trimming it to the desired shape. Then I remove the model and build the ground porcelain to the same contour.

If the inlay is a small one, I usually bake it twice; once to biscuit the Close body and once to vitrify the coloring or enamel body.

If large, I biscuit the Close body, then barely fuse a layer of colored body and then vitrify the enamel body.

I believe that as soon as a satisfactory cement is discovered, the inlay will, to a large extent, supplant gold as a filling material.

